# Emerging Roles of NANOS RNA-Binding Proteins in Cancer

**DOI:** 10.3390/ijms23169408

**Published:** 2022-08-20

**Authors:** Erkut Ilaslan, Marcin Piotr Sajek, Jadwiga Jaruzelska, Kamila Kusz-Zamelczyk

**Affiliations:** 1 Institute of Human Genetics, Polish Academy of Sciences, 60-479 Poznan, Poland; 2 RNA Bioscience Initiative, University of Colorado School of Medicine, Aurora, CO 80045, USA; 3 Department of Biochemistry and Molecular Genetics, University of Colorado School of Medicine, Aurora, CO 80045, USA

**Keywords:** NANOS1, NANOS2, NANOS3, cancer, RNA-binding protein

## Abstract

In recent years, growing evidence demonstrates that mammalian Nanos RNA-binding proteins (Nanos1, Nanos2, and Nanos3), known for their indispensable roles in germline development, are overexpressed in a variety of cancers. This overexpression contributes to various oncogenic properties including cancer growth, invasiveness, and metastasis. Here, we highlight recent findings regarding the role of mammalian Nanos RNA-binding proteins and the mechanisms of their overexpression in cancer. In addition, we present expression profiles of human *NANOS* genes and their oncogenic transcriptional regulators obtained from publicly available cancer and normal tissue RNA-Seq datasets. Altogether, we emphasize the functional significance of NANOS proteins across human cancers as well as highlight the missing links to understanding the full scope of their role in carcinogenesis.

## 1. Introduction

The development of multicellular organisms is accomplished by the specification, differentiation, and organization of cells into specialized lineages that compose organs and tissues. This process leads to a terminal, differentiated, and non-proliferative stage of a given cell. One of the hallmarks of cancer includes disruptions in developmental processes, enabling the maintenance or emergence of immature stem-like cells with high uncontrolled proliferative potential [1]. Organismal development and cellular differentiation are coordinated by a complex spatio-temporal control of gene expression, including an array of transcriptional and post-transcriptional mechanisms. Therefore, the overexpression of transcription factors or RNA-binding proteins involved in post-transcriptional developmental processes can lead to the reprogramming of cells to a stem-like state that gives rise to cancer [2]. 

RNA-binding proteins (RBPs) are essential components of post-transcriptional gene regulation that play critical roles in every step of this process. Growing evidence shows that a broad range of RBPs, involved in different stages of post-transcriptional regulation, carry out cancer-related functions [3,4]. Thus, RBPs became an attractive subject of cancer research. Many of these RBPs demonstrate abnormal expression [5] and harbor mutations in human cancers [6]. Furthermore, RBP expression profiles are used to build prognostic models of human cancer [7]. Thus, growing evidence highlights the emerging importance of RBPs and post-transcriptional regulation in cancer. 

Nanos proteins are highly conserved RBPs that play crucial roles in germline development throughout the animal kingdom, for example, in poriferans [8], echinoderms [9], cnidarians [10], annelids [11], arthropods and all vertebrates [12,13]. The first Nanos protein was characterized in *Drosophila* for its importance in posterior body patterning. This discovery contributed to the Nobel Prize in 1995 for understanding early developmental events in animals [14] and pioneered the research required to elucidate the functions of Nanos proteins in the animal kingdom. One of the initial findings was that the nanos protein is specifically expressed in the abdomen and plays a role of a morphogen necessary in *Drosophila* abdomen patterning. Together with the pumilio protein, nanos repress the expression of hunchback, an anterior morphogen. This repression is initiated by pumilio, which recognizes and binds nucleotide motif in the 3’UTR of *hunchback* mRNA, initially called Nanos Response Element (NRE) [15] and later renamed as Pumilio Binding Element (PBE) [16]. Upon binding to PBE, pumilio protein recruits nanos, which is followed by repression and degradation of *hunchback* mRNA in the posterior. This and the following findings led to the understanding that pumilio’s role is to select the RNA target for repression while nanos itself is a cofactor. Thus, further research was focused on Pumilio proteins as they were perceived as the main component of this type of post-transcriptional regulation. However, in comparison to *Pumilio* knockout models, *Nanos* knockout in model organisms demonstrated more severe developmental phenotypes such as infertility [17,18]. Moreover, recent studies showed that Nanos proteins can directly bind to RNA by recognizing specific motifs [19,20], suggesting that these proteins can carry out important functions independent of recruitment by Pumilio.

**Figure 1:**
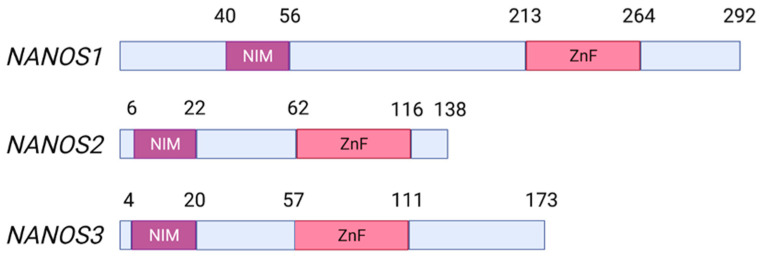
Schematic representation of human *NANOS* genes showing functional Not1 Interacting Motif (NIM) and (CHCC)2 zinc finger (ZnF) domains. Created with Biorender.com (accessed on 15 July 2022).

Nanos proteins act as post-transcriptional regulators by binding to target mRNAs followed by the recruitment of Ccr4-Not deadenylase complex for repression. All Nanos proteins contain a C-terminal Nanos-type (CCHC)_2_ zinc finger RNA-binding domain. Moreover, all vertebrate Nanos proteins contain Not1-Interacting Motif (NIM) (Figure 1). In turn, *Drosophila* Nanos contains Nanos Effector Domain (NED) that binds Not-1 and Not-3 components of the Ccr4–Not complex [21,22]. 

Nanos proteins play indispensable roles in germline development by mainly regulating proliferation, cell cycle, and apoptosis [23]. By regulating these processes, Nanos proteins are involved in the maintenance and differentiation of germline stem cells [23,24,25]. With the emergence of multiple Nanos paralogues during evolution, mammalian Nanos paralogues (Nanos1, Nanos2, and Nanos3) diverged in their functions and adopted distinct roles in germline development. For example, NANOS1 represses apoptosis in human germ cell line TCam-2 [26], while mouse Nanos2 is crucial for inducing mitotic arrest during male germ cell development [27]. Lastly, Nanos3 is important for the maintenance of the male germline stem cells [28] and suppresses premature spermatogonial differentiation [29]. Thus, the dysregulation of Nanos proteins disrupts proliferation, cell cycle, apoptosis, and differentiation, processes that are highly connected with carcinogenesis.

In recent years, growing evidence shows that NANOS proteins are overexpressed in a variety of human cancers and regulate mRNAs to exert oncogenic properties. Here, we take a closer look at the emerging role of NANOS RNA-binding proteins in cancer. We discuss recent studies demonstrating their roles in human cancers and potential mechanisms that induce their overexpression. Furthermore, to complement the literature, we provide a bioinformatic comparison of expression profiles of human NANOS genes and their oncogenic transcriptional regulators obtained from publicly available expression datasets of cancer (TCGA) and normal tissues (GTEx).

## 2. NANOS1 in Cancer

The first report for the potential role of NANOS proteins in various types of cancer was published regarding the correlation between the expression of *NANOS1* and tumor suppressor gene *CDH1*-encoding Epithelial cadherin (E-cadherin) [30]. E-cadherin is a protein acting as a cell–cell adhesion molecule. The loss of its expression is associated with tumor cell migration stimulation and metastasis [31]. It was shown that the exogenous expression of *CDH1* in a human breast cancer cell line MDA-MB-231 resulted in a decrease in *NANOS1* mRNA levels. Although it is not clear how *CDH1* expression results in a decrease in *NANOS1* level, this negative correlation between *NANOS1* and *CDH1* expression on RNA level is observed also in multiple cancer cell lines, representing breast, colon, brain, skin, and eye cancers. Specifically, in cell lines expressing low levels of *CDH1*, high levels of *NANOS1* mRNA were observed. Interestingly, the induced expression of NANOS1 in a colorectal cancer cell line DLD1 resulted in the abolishment of E-cadherin-dependent cell–cell adhesion and demonstrated strong migratory and invasive properties [30]. In a follow-up study, it was shown that *NANOS1* is overexpressed in invasive lung carcinomas, which cause an upregulation of MMP14 (MT1-MMP) on both RNA and protein levels. MMP14 is an endopeptidase that breaks down extracellular matrices under normal physiological conditions. The overexpression of this protein is correlated with the malignant progression of many human cancers, including lung and breast cancers [32]. However, from these studies, it is not clear how NANOS1 regulates E-cadherin and MMP14, for example, whether their mRNAs are direct post-transcriptional targets of NANOS1. Regardless, these studies link the overexpression of NANOS1 to the high susceptibility of metastasis by increasing the invasiveness of cancer cells.

Testicular seminoma is the most prominent type of human testis germ cell tumor, most frequently occurring in young men. The role of NANOS1 in that pathological context was studied in the seminoma TCam-2 cell line. These cells represent human germ cells at an early stage of germ cell development, shortly after they populate primary gonads. This study showed that the overexpression of NANOS1 led to an increase in the proliferation of TCam-2 cells. Additionally, it demonstrated that NANOS1 caused a significant decrease in apoptosis. It was further demonstrated by transcriptomic data that the NANOS1-mediated decrease in apoptosis was accompanied by the downregulation of pro-apoptotic genes such as *RHOB*, *MYC*, and *GADD45* family genes [26]. These genes are downstream targets of p53 and are involved in p53-mediated DNA-damage checkpoint pathway. In conclusion, this study shows the anti-apoptotic function of NANOS1 by regulating genes involved in the p53-mediated DNA-damage checkpoint pathway as well as its pro-proliferative effect in testis cancer. 

In the last decade, microRNAs (miRNAs) have been extensively studied for their potential role in cancer. These short RNA molecules can act as onco-suppressors by targeting the mRNA of oncogenes or, the opposite, play a pro-oncogenic role by targeting mRNAs of onco-suppressors. As such, they can be utilized as stable biomarkers that can be found in patient blood samples and, therefore, can be used for diagnostic purposes. A recent study demonstrated that miR-127 is downregulated in triple-negative breast cancer. Authors showed that the exogenous expression of miR-127 reduced the growth and metastatic potential of triple-negative breast cancer cells [33]. Moreover, high levels of miR-127 expression are correlated with a higher rate of patient survival. This study identified *NANOS1* as a target of miR-127 that downregulates *NANOS1* expression. Although this study demonstrates that miR-127 also downregulates other genes that are overexpressed in cancer, such as *FOXO6*, *SOX11*, *SOX12*, and *FASN*, it provides the first example that targeting NANOS1 could be a potential cancer treatment strategy. Using specific antisense oligos targeting NANOS1 in cancers which it is overexpressed is required to explore whether this could be a viable approach.

**Figure 2:**
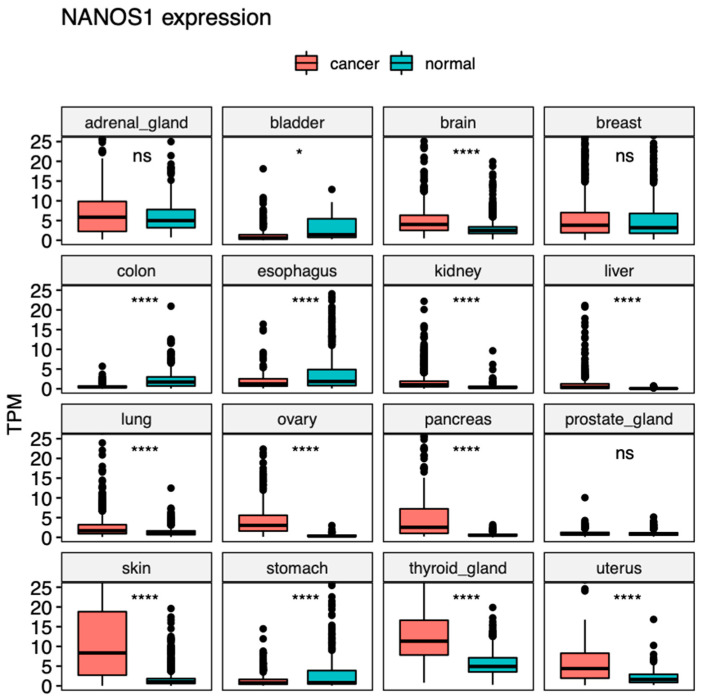
NANOS1 expression across human cancers. Box-plot comparison of transcripts per million (TPM) expression values obtained from RNA-Seq experiments in cancer (TCGA) (red boxes) and normal tissues (GTEx) (blue boxes). Statistical significance was determined using a two-tailed *t*-test. ns = non-significant, * = *p* < 0.05, **** = *p* < 0.0001.

**Figure 3:**
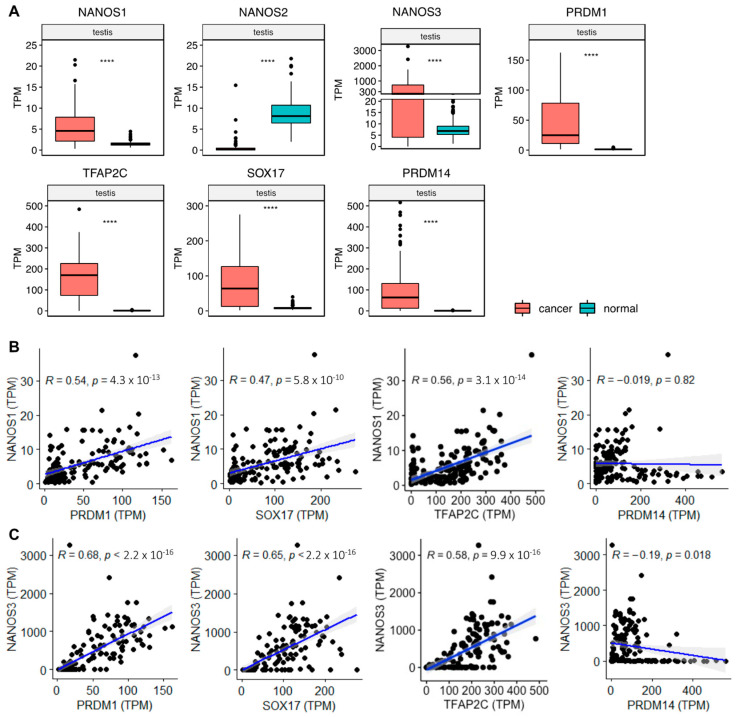
Expression of NANOS proteins and their upstream transcriptional program in testis cancer. (**A**) Box-plot comparison of transcripts per million (TPM) expression values obtained from RNA-Seq experiments in testis cancer (TCGA) (red boxes) and normal testis tissues (GTEx) (blue boxes). Statistical significance was determined using a two-tailed *t*-test. **** = *p* < 0.0001. (**B**) NANOS1 and (**C**) NANOS3 TPM expression correlation with their upstream transcriptional program in testis cancer tissues obtained from TCGA. *R* = correlation coefficient. *p* = *p*-value.

Our bioinformatic analysis of publicly available expression datasets for cancer (TCGA) and normal tissues (GTEx) showed that *NANOS1* is overexpressed in a variety of human cancers, including brain, kidney, liver, lung, ovary, pancreas, skin, thyroid gland uterus, and testis cancers (Figure 2 and Figure 3A). On the other hand, *NANOS1* expression is diminished in colon, esophagus, and stomach cancers. In the case of the majority of these tissues, how the differential expression of *NANOS1* could contribute to carcinogenesis remains unknown. 

## 3. NANOS2 in Cancer

NANOS2 protein is expressed only in male germ cells, and it is implicated in the male sexual determination of the germline. Nanos2 is an important factor to induce mitotic arrest of mouse germ cells during development [34]. In mammals, the mitotic arrest of male germline is required for male sexual commitment and its timing (which is regulated by Nanos2) is crucial for male sex development. A recent study showed that Nanos2 induces a mitotic arrest of mouse embryonic germ cells by repressing Rheb, a protein that binds to the mTORC1 complex and activates its kinase activity, thus acting as a regulator of mTORC1 [27]. mTORC1 is one of the two mTOR complexes that are present in mammals and it is highly implicated in cancer due to its essential role in regulating cellular metabolism and proliferation. Two recent studies linked the abnormal expression of Nanos2 to testis cancer incidence. It was shown in mice that the delayed expression of Nanos2 increases teratoma formation susceptibility of the germ cells [35]. Another study showed that during germ cell development in mice, a subpopulation of germ cells lacking Nanos2 expression maintains a pluripotent state. These cells are characterized by the overexpression of Myc, E2f, mTOR signaling, and G2/M phase genes such as *Plk1* and *Cdk1.* These cells give rise to embryonal carcinoma [36]. These data suggest that the lack of NANOS2 potentially abolishes the ability of the cell to downregulate oncogenes such as *MYC* and *E2F* family members as well as mTOR-signaling pathway genes. This indicates that a lack of NANOS2 protein increases the susceptibility of testis cancer. 

In line with the above findings, our analysis of publicly available expression datasets of cancer (TCGA) and normal tissues (GTEx) revealed that the level of *NANOS2* diminished in testis cancer compared to normal testis tissues (Figure 3A). Moreover, unlike NANOS1 and NANOS3, NANOS2 did not show any overexpression patterns in human cancers. Collectively, these studies show that NANOS2 downregulation in male germ cells has a pro-mitotic effect and gives rise to germ cell cancers.

## 4. NANOS3 in Cancer

Mammalian Nanos3 is expressed in primordial germ cells (PGCs), beginning from their specification through their migration into the gonads and maintains its expression in the gonadal germ cells. Interestingly, the potential role of Nanos3 in PGCs migration is in accordance with research, showing its implication in tumor invasiveness and metastasis. 

Non-small cell lung carcinomas are the most common type that consists of around 80% of all lung cancers and are the leading cause of cancer mortality [37]. It was shown that NANOS3 is overexpressed and is a marker of this type of cancer [38]. The authors of this study identified *VIM* mRNA as a direct and *CDH1 mRNA* as an indirect target of NANOS3. VIM and E-cadherin proteins are involved in epithelial–mesenchymal transition. This suggests that the overexpression of NANOS3 contributes to the invasiveness of this type of cancer. The authors suggest that *CDH1* is potentially regulated by NANOS3 at the transcriptional level due to a lack of evidence on its direct regulation at the post-transcriptional level. However, the mechanism of how *CDH1* is regulated by NANOS3 remains elusive. 

Another study showed the overexpression of NANOS3 in glioblastoma cell lines and tissues. Glioblastoma is the most common type of brain cancer that is characterized by high heterogeneity, mortality, and short median survival rate [39]. Moreover, molecular mechanisms leading to glioblastoma are not well understood. In a study, the CRISPR-Cas9 knockdown of NANOS3 in glioblastoma cell lines resulted in reduced proliferation, migration, and invasiveness, suggesting a role for NANOS3 in this disease. Moreover, xenografting NANOS3 knockdown cells obtained from glioblastoma cell lines into mice in comparison to control xenografts significantly reduced the tumor’s growth [40]. Further research on how NANOS3 overexpression contributes to the pathogenesis of glioblastoma would lead to a better understanding of this disease. 

**Figure 4:**
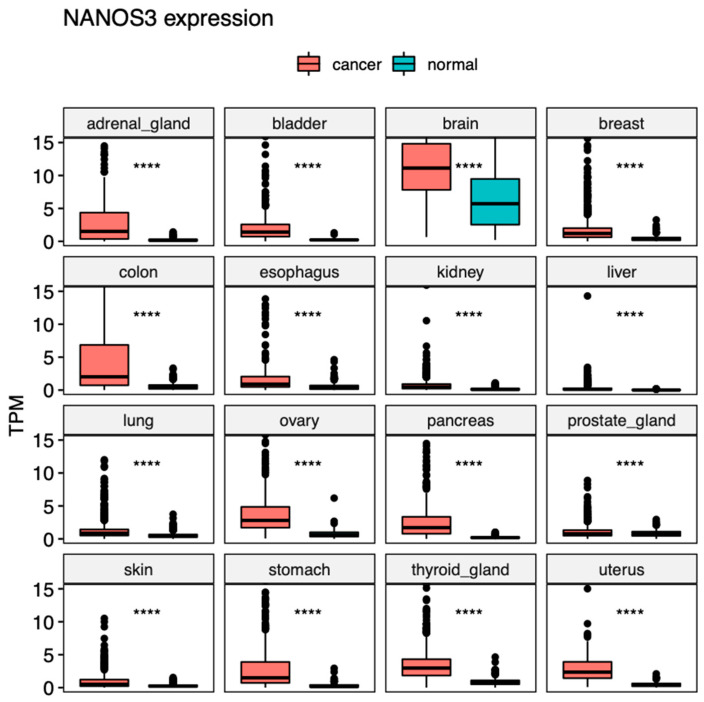
NANOS3 expression across human cancers. Box-plot comparison of transcripts per million (TPM) expression values obtained from RNA-Seq experiments in cancer (TCGA) (red boxes) and normal tissues (GTEx) (blue boxes). Statistical significance was determined using a two-tailed *t*-test. **** = *p* < 0.0001.

Collectively, these studies show that the overexpression of NANOS3 in cancers is highly linked with an increase in tumor invasiveness, metastasis, and tumor growth. Our bioinformatic analysis of publicly available expression datasets of cancer (TCGA) and normal tissues (GTEx) showed that NANOS3 is overexpressed in all cancers derived from the 17 human tissues, suggesting its role in driving uncontrolled cell proliferation, invasiveness, and metastasis that could play a role in these types of cancers (Figure 3A and Figure 4). In line with the literature discussed above, NANOS3 is overexpressed in brain and lung cancers in comparison to normal tissues. Moreover, in contrast to NANOS1, NANOS3 is not expressed in normal tissues except in testis and brain tissues, suggesting that it could be used as a marker for a broad range of cancers and, thus, is an attractive gene for diagnostic purposes in the future.

## 5. Dysregulation of NANOS Transcriptional Regulators in Cancer

The expression profile of NANOS proteins during germ cell development is well-described in model organisms. However, at what stage these proteins are expressed during human germ cell development and the upstream transcriptional program that regulates their expression remained unknown due to ethical constraints of research on human embryos. Recent studies shed light on how NANOS protein expression is regulated during germ cell development, thus contributing to the understanding of how these proteins can be overexpressed in cancer. 

A comprehensive study analyzing the gene expression across mammalian development, including humans, provided insight into the timing of NANOS expression in human germ cell development [41]. According to this study, in human testis, NANOS1 starts to be expressed on the 5th, NANOS2 on the 10th and NANOS3 on the 6th week post conception. In human ovaries, NANOS1 starts to be expressed on the 6th, NANOS2 is only expressed briefly on the 10th, and NANOS3 starts to be expressed on the 4th week post conception.

In support of this, emerging studies in human PGC-like models provided crucial data on the early genetic programming of human PGCs specification. These studies revealed some of the transcription factors (TFs) important for germ cell specification, functioning upstream of NANOS proteins, and their expression patterns throughout differentiation. In one of these studies, the authors used human-induced pluripotent stem cells (hiPSC) and differentiated these cells into human primordial germ cell-like cells (hPGCLCs). During this differentiation process, thirteen TFs’ binding motifs were significantly enriched in upregulated genes. One of these TFs is TFAP2C and its binding motif is present upstream of the *NANOS3* [42]. Interestingly, Tfap2c binds to the promoter of Nanos3 in mouse germ cells and Tfap2c-deficient germ cells demonstrate deregulation in epigenetic remodeling, cell cycle, and pluripotency genes. Mice with heterozygous deletion of both Tfap2c and Nanos3 develop germ cell cancer [43]. Similarly to NANOS3 overexpression in cancer, TFAP2C overexpression was also reported in various cancer types [44]. Taken together, these studies demonstrate a potential mechanism of TFAP2C-mediated overexpression of NANOS3 in cancer.

A recent study showed that PRDM14 acts as a transcriptional activator for both NANOS1 and NANOS3 in hPGCLCs [45]. Interestingly, PRDM14 was also shown to be upregulated in a broad range of cancer types [46,47,48]. However, to what extent the carcinogenesis induced by overexpression of TFs upstream of NANOS proteins such as PRDM14 and TFAP2C is linked to the downstream overexpression of NANOS proteins remains unexplored. To address this, we analyzed the correlation between NANOS1 and NANOS3 with its upstream transcriptional program in human primordial germ cells including transcription factors PRDM1, SOX17, TFAP2C, and PRDM14 expression in testis cancer samples (Figure 3B,C). Our results showed that both NANOS1 and NANOS3 expressions have a positive correlation with PRDM1, SOX17, and TFAP2C. On the other hand, PRDM14 did not show any statistically significant correlation with NANOS1 and a weak negative correlation with NANOS3. However, in the last case, many samples with very low to no NANOS1 and NANOS3 expression contribute to these correlations, whereas a positive correlation is observed in samples with overexpression of both NANOS1 and NANOS3. Therefore, PRDM14 likely unable to induce the expression of these proteins alone and requires other factors such as PRDM1, SOX17, and TFAP2C to contribute to NANOS1 and NANOS3 overexpression. These results suggest a potential contribution to the overexpression of NANOS1 and NANOS3 by PRDM1, SOX17, TFAP2C, and PRDM14 in the context of human testis cancer.

One example of the direct involvement of NANOS transcriptional regulation in cancer is described in a study demonstrating pRb and E2F transcription factors acting together as a transcriptional repressor complex for NANOS proteins. pRb is encoded by *RB1*, a tumor suppressor gene, and its expression is diminished in a broad range of cancers [49]. The E2F family of transcription factors comprises crucial regulators of the cell cycle. These TFs induce the expression of the G1/S phase genes involved in cellular growth and DNA synthesis (for review see [50]) thus playing a central role in the cell cycle. The overexpression of these transcription factors induces proliferation and is implicated in cancer [51]. Authors show that, upon binding to the E2F family of transcription factors, pRb acts as a transcriptional repressor complex for NANOS proteins. Thus, the downregulation of pRb leads to the upregulation of NANOS proteins [52]. This upregulation leads to a repression of pro-apoptotic genes, thus potentially contributing to carcinogenesis. A different example includes the suppression of NANOS1 expression by HDAC3 in non-small cell lung cancer cells. HDAC3 is a histone deacetylase protein that functions by removing an acetyl group from the histones, thus increasing the positive charge of histone and its affinity to negatively charged DNA. This increase in affinity results in the tighter formation of nucleosomes and leads to an inaccessibility of naked DNA and reduced gene expression from a given region. In this study, the authors demonstrate that KDM2A represses HDAC3 by removing methyl groups from dimethylated H3K36 at the *HDAC3* promoter. KDM2A, also known as FBXL11, is a lysine demethylase protein that removes methyl groups from H3 proteins. HDAC3 binds to the proximity of cell cycle genes such as *CDK6* as well as *NANOS1* and downregulates their expression. This study suggests that KDM2A overexpression enhances cancer progression and metastasis by downregulating HDAC3, which in turn results in the overexpression of NANOS1 [53]. Taken together, these studies demonstrate various mechanisms of how the overexpression of NANOS proteins can be achieved in a broad range of cancer cells.

FOXM1 is a transcription factor known for its vital role in regulating proliferation. This transcription factor is necessary for development in mammals by inducing organismal growth and by promoting proliferation. Moreover, it is a well-known oncogene. FOXM1 was shown to bind to the promoter of *NANOS3* in a human germ cell model [54]. This study showed a mechanism in which NANOS3 expression is induced by FOXM1; in return, NANOS3 represses FOXM1 through 3’UTR of its mRNA. Moreover, FOXM1 and NANOS3 expressions are negatively correlated in human testis cancer. However, it is not clear exactly how this mechanism contributes to cancer. One possible explanation is that NANOS3-FOXM1 axis dysregulation leads to abnormal germ cell development by disrupting the cell cycle. As a result, abnormal germ cells may predispose to carcinogenesis (similarly to Nanos2 downregulation in increased testis cancer incidence discussed above).

## 6. Future perspectives

NANOS proteins interact and form post-transcriptional repressor ribonucleoprotein (RNP) complexes with other RBPs, which are also implicated in cancer. Recently, it was reported that mouse Dnd1 is a critical partner of both Nanos2 and Nanos3 proteins in increasing the susceptibility of teratoma formation of PGCs [55]. Moreover, PUM proteins, one of the best examples of NANOS partner RBPs, were shown to be overexpressed in many types of cancer [56]. It is likely that the NANOS interactome extends beyond DND1, PUM proteins, and CCR4-NOT deadenylase complexes. Therefore, in order to gain an improved insight into the role of NANOS proteins in cancer, the identification of complete Nanos protein ribonucleoprotein interactome in normal cells, as well as in different cancers, is necessary. The identification of such NANOS interactomes by using methods such as immunoprecipitation followed by mass spectrometry (IP-MS) could provide further insights into their functions. Particularly in the cellular context of cancer, the overexpression of NANOS proteins might enable the formation of cancer-specific RNP complexes that are physiologically not viable in healthy cells. Further studies exploring these aspects could reveal potential Nanos-dependent vulnerabilities that can be exploited for cancer treatment. 

Most studies that we discussed in this paper do not address the mechanism of how Nanos proteins recognize and regulate their target mRNAs encoding proteins with oncogenic properties. Thus, whether Nanos proteins regulate these targets by direct RNA binding requires clarification. Moreover, unlike NANOS2 and NANOS3, NANOS1 direct RNA-binding has not been shown yet. Studies taking advantage of state-of-the-art crosslinking and immunoprecipitation (CLIP) approaches such as eCLIP [57] to characterize direct NANOS RNA targets are required to understand the full extent of their role in cancer. 

Our bioinformatic analysis suggests that the overexpression of NANOS1 and NANOS3 in testis cancer is potentially achieved by the upregulation of their upstream transcriptional program. However, the mechanism of NANOS overexpression in other types of cancers is so far unexplored. Further studies elucidating how this overexpression is achieved could lead to new therapeutic strategies in a variety of cancers.

Intriguingly, two independent studies showed that the NANOS3 protein is localized in the nucleus and suggest a nuclear function for this protein known for its cytoplasmic role as a post-transcriptional repressor. In one of these studies, it was shown that in highly proliferative human germ cells within the seminiferous tubules, NANOS3 is co-localized with chromosomal DNA inside the nucleus [58]. Another study showed that in non-small cell lung carcinomas, NANOS3 is localized in both the nucleus and cytoplasm [38]. These studies show that in highly proliferative cells, NANOS3 localizes in the nucleus, potentially contributing to proliferation. However, whether NANOS3, additionally to its role in post-transcriptional regulation, can potentially also bind to DNA and act as a transcriptional regulator remains elusive. Given that zinc finger domains are known to bind to DNA, it is possible that the zinc finger domain of Nanos proteins potentially facilitates binding to DNA. Such binding was previously shown for other proteins containing tandem CCHC domains [59,60]. Further research is necessary to elucidate the potential involvement of Nanos proteins in transcriptional regulation and its implications for cancer. 

## Data Availability

The expression datasets as well as the code used for analysis and visualization are available upon request from the corresponding authors.
